# Supplementary material for: Supporting reanalysis and reuse of clinical trial data: a case study
Source: Trials. 2026 Mar 25;27:351. doi: 10.1186/s13063-026-09646-y (PMC13137473; doi:10.1186/s13063-026-09646-y)
Supplement: Supplementary file 1 — Supplementary Material 1. [file 13063_2026_9646_MOESM1_ESM.pdf]

Supplementary material for paper *Supporting Reanalysis and Reuse of Clinical Trial Data: A Case Study*

Supplementary Table 1: Comparison on log-rank test and Cox proportional hazard model for disease-free survival (DFS) in CAO/ARO/AIO-04 trial 1

Supplementary Table 2: Numeric comparison of table 1 (participant characteristics) in CAO/ARO/AIO-04 trial with team reproductions 2

Supplementary table 3. Numeric comparison of table 2 (first events for primary endpoint disease-free survival) in CAO/ARO/AIO-04 trial with team reproductions 5

Supplementary table 4. Numeric comparison of table 3 (all-cause deaths) in CAO/ARO/AIO-04 trial with team reproductions 6

Supplementary table 5. Evaluation on the reproduced paper according to CONSORT 2010 checklist 7

Supplementary Table 1: Comparison on log-rank test and Cox proportional hazard model for disease-free survival (DFS) in CAO/ARO/AIO-04 trial

	Original	Team 1	Team 2	Team 3	Team 4	Team 5*
**Log-rank test**	0.03	0.034	0.03	0.03418	/	0.0319
**From Cox model**						
**Hazard ratio**	0.79	0.79	0.79	0.7939123	0.796107087246087	0.80 †
**95% confidence interval**	0.64-0.98	0.64-0.98	0.64-0.98	/	0.646134917224772 - 0.980888785713113	0.65-0.99 †
***P*-value**	0.03	0.03	0.03	0.03	/	0.0369 †

Numbers are highlighted in red if the result was not reproduced in the datathon.

* Analysis was run in Python in this team. Analyses from other teams were run in R.

/ Analysis/statistics was not considered.

† Stratified Cox regression was used due to the package availability. Mixed-effect Cox model was used in the analyses from the other teams.

Supplementary Table 2: Numeric comparison of table 1 (participant characteristics) in CAO/ARO/AIO-04 trial with team reproductions

	Original		Team 1		Team 2		Team 3		Team 4		Team 5*	
	Investi-gational (n=613)	Control (n=623)	Investi-gational (n=613)	Control (n=623)	Investi-gational (n=613)	Control (n=623)	Investi-gational (n=613)	Control (n=623)	Investi-gational (n=613)	Control (n=623)	Investi-gational (n=613)	Control (n=623)
**Age (years)**												
**Mean (SD)**	62 (10)	62 (10)	62 (10)	62 (10)	62 (±10)	62 (±10)	62	62	62	62	62 (10)	62 (10)
**Median (IQR)**	64 (55–70)	63 (55–70)	64 (55-70)	64 (55-70)	64 (55–70)	64 (55–70)	64 (55-70)	64 (55-70)	64 (55–70)	64 (55–70)	64 (55-70)	64 (55-70)
**Sex**												
**Male**	434 (71%)	440 (71%)	434 (71%)	440 (71%)	434 (71%)	440 (71%)	434 (71%)	440 (71%)	874 §	§	434 (71%)	440 (71%)
**Female**	179 (29%)	183 (29%)	179 (29%)	183 (29%)	179 (29%)	183 (29%)	179 (29%)	183 (29%)	362 §	§	179 (29%)	183 (29%)
**ECOG performance status**												
**0**	483 (79%)	475 (76%)	483 (79%)	475 (76%)	483 (79%)	475 (76%)	483 (79%)	475 (76%)	958 §	§	483 (79%)	475 (76%)
**1-2**	123 (20%)	141 (23%)	123 (20%)	141 (23%)	123 (20%)	141 (23%)	123 (20%)	141 (23%)	264 §	§	123 (20%)	141 (23%)
**Missing**	7 (1%)	7 (1%)	7 (1%)	7 (1%)	7 (1%)	7 (1%)	7 (1%)	7 (1%)	/	/	7 (1%)	7 (1%)
**Clinical T category**												
**cT2**	22 (4%)	32 (5%)	22 (4%)	32 (5%)	22 (4%)	32 (5%)	22 (4%)	32 (5%)	1140 §†	§†	22 (4%)	32 (5%)
**cT3**	549 (90%)	537 (86%)	549 (90%)	537 (86%)	549 (90%)	537 (86%)	549 (90%)	537 (86%)	§†	§†	549 (90%)	537 (86%)
**cT4**	41 (7%)	50 (8%)	41 (7%)	50 (8%)	41 (7%)	50 (8%)	41 (7%)	50 (8%)	91§	§	41 (7%)	50 (8%)
**Missing**	1 (<1%)	4 (<1%)	1 (<1%)	4 (<1%)	1 (0%)	4 (1%)	1 (<1%)	4 (<1%)	/	/	1 (<1%)	4 (<1%)
**Clinical N category**												
**cN0**	146 (24%)	159 (26%)	146 (24%)	159 (26%)	146 (24%)	159 (26%)	146 (24%)	159 (26%)	305 §	§	146 (24%)	159 (26%)
**cN1-2**	452 (74%)	451 (72%)	452 (74%)	451 (72%)	452 (74%)	451 (72%)	452 (74%)	451 (72%)	903 §	§	452 (74%)	451 (72%)
**Missing**	15 (2%)	13 (2%)	15 (2%)	13 (2%)	15 (2%)	13 (2%)	15 (2%)	13 (2%)	/	/	15 (2%)	13 (2%)

	Original		Team 1		Team 2		Team 3		Team 4		Team 5*	
	Investi-gational (n=613)	Control (n=623)	Investi-gational (n=613)	Control (n=623)	Investi-gational (n=613)	Control (n=623)	Investi-gational (n=613)	Control (n=623)	Investi-gational (n=613)	Control (n=623)	Investi-gational (n=613)	Control (n=623)
**Clinical disease stage**												
**Stage II**	146 (24%)	159 (26%)	146 (24%)	159 (26%)	146 (24%)	159 (26%)	146 (24%)	159 (26%)	302 §	§	146 (24%)	159 (26%)
**Stage III**												
**cT1-2 N1-2**	22 (4%)	32 (5%)	22 (4%)	32 (5%)	22 (4%)	32 (5%)	22 (4%)	32 (5%)	/	/	22 (4%)	32 (5%)
**cT3-4 N1-2**	430 (70%)	419 (67%)	430 (70%)	419 (67%)	430 (70%)	419 (67%)	430 (70%)	419 (67%)	/	/	430 (70%)	419 (67%)
**Missing**	15 (2%)	13 (2%)	15 (2%)	13 (2%)	15 (2%)	13 (2%)	15 (2%)	13 (2%)	/	/	15 (2%)	13 (2%)
**Location from anal verge**												
**0-5cm**	249 (41%)	216 (35%)	249 (41%)**	216 (35%)**	249 (41%)	216 (35%)	249 (41%)	216 (35%)	465 §	§	249 (41%)**	216 (35%)**
**>5-10cm**	302 (49%)	336 (54%)	302 (49%)**	336 (54%)**	302 (49%)	336 (54%)	302 (49%)	336 (54%)	638 §	§	302 (49%)**	336 (54%)**
**>10cm**	55 (9%)	64 (10%)	55 (9%)	64 (10%)	55 (9%)	64 (10%)	55 (9%)	64 (10%)	119 §	§	55 (9%)	64 (10%)
**Missing**	7 (1%)	7 (1%)	7 (1%)	7 (1%)	7 (1%)	7 (1%)	7 (1%)	7 (1%)	/	/	7 (1%)	7 (1%)
**Histology**												
**Adenocarcinoma**	599 (98%)	597 (96%)	599 (98%)	597 (96%)	599 (98%)	597 (96%)	599 (98%)	597 (96%)	/	/	599 (98%)	597 (96%)
**Mucinous adenocarcinoma**	5 (<1%)	11 (2%)	5 (<1%)	11 (2%)	5 (1%)	11 (2%)	5 (<1%)	11 (2%)	/	/	5 (<1%)	11 (2%)
**Signet-ring cell carcinoma**	3 (<1%)	4 (<1%)	3 (<1%)	4 (<1%)	3 (0%)	4 (1%)	3 (<1%)	4 (<1%)	/	/	3 (<1%)	4 (<1%)
**Other or missing**	6 (1%)	11 (2%)	6 (<1%)	11 (2%)	6 (1%)	11 (2%)	6 (1%)	11 (2%)	/	/	6 (<1%)	11 (2%)
**Tumour differentiation**												
**Well differentiated (G1)**	33 (5%)	31 (5%)	33 (5%)	31 (5%)	33 (5%)	31 (5%)	33 (5%)	31 (5%)	/	/	33 (5%)	31 (5%)
**Moderately differentiated (G2)**	496 (81%)	502 (81%)	496 (81%)	502 (81%)	496 (81%)	502 (81%)	496 (81%)	502 (81%)	/	/	496 (81%)	502 (81%)
**Poorly diff**	49 (8%)	50 (8%)	49 (8%)	50 (8%)	49 (8%)	50 (8%)	49 (8%)	50 (8%)	/	/	49 (8%)	50 (8%)

	Original		Team 1		Team 2		Team 3		Team 4		Team 5*	
	Investi-gational (n=613)	Control (n=623)	Investi-gational (n=613)	Control (n=623)	Investi-gational (n=613)	Control (n=623)	Investi-gational (n=613)	Control (n=623)	Investi-gational (n=613)	Control (n=623)	Investi-gational (n=613)	Control (n=623)
**erentiated (G3)**												
**Missing data**	35 (6%)	40 (6%)	35 (6%)	40 (6%)	35 (6%)	40 (6%)	35 (6%)	39 (6%)	/	/	35 (6%)	40 (6%)

Count (percentage) is reported in the table if not specified otherwise. Numbers are highlighted in red if the result was not reproduced during the datathon.

* Analysis was run in Python in this team. Analyses from the other teams were run in R.

§: The results were calculated for both investigational and control arms combined.

/ Analysis/statistics was not considered.

† The data was summarized for cT2-3 across intervention arms.

** The categories are noted as <6cm and 6-10cm in the reproduction.

Supplementary table 3. Numeric comparison of table 2 (first events for primary endpoint DFS) in CAO/ARO/AIO-04 trial with team reproductions

	Original		Team 1		Team 2		Team 3		Team 4		Team 5*	
	Investiga-tional (n=613)	Control (n=623)	Investiga-tional (n=613)	Control (n=623)	Investiga-tional (n=613)	Control (n=623)	Investiga-tional (n=613)	Control (n=623)	Investiga-tional (n=613)	Control (n=623)	Investiga-tional (n=613)	Control (n=623)
**Macroscopically incomplete local resection (R2)**	4 (<1%)	9 (1%)	4 (<1%)	9 (1%)	4 (1%)	9 (1%)	5 (1%)	10 (2%)	/	/	4 (<1%)	9 (1%)
**Locoregional recurrence (after R0/R1 resection)**												
**As first event**	12 (2%)	23 (4%)	12 (2%)	23 (4%)	12 (2%)	23 (4%)	12 (2%)	23 (4%)	1	7	12 (2%)	23 (4%)
**Cumulative**	18 (3%)	38 (6%)	12 (2%)	25 (4%)	/	/	/	/	12	25	18 (3%)	38 (6%)
**Distant metastasis or progression**												
**As first event**	107 (17%)	140 (22%)	107 (17%)	140 (22%)	107 (17%)	140 (22%)	107 (17%)	140 (22%)	50	59	107 (17%)	140 (22%)
**Cumulative**	118 (19%)	151 (24%)	118 (19%)	151 (24%)	118 (19%)	151 (24%)	/	/	/	/	118 (19%)	151 (24%)
**Death as first event**	36 (6%)	26 (4%)	36 (6%)	26 (4%)	36 (6%)	26 (4%)	36 (6%)	26 (4%)	49	51	36 (6%)	26 (4%)
**First event for disease-free survival (total)**	159 (26%)	198 (32%)	159 (26%)	198 (32%)	159 (26%)	198 (32%)	/	/	/	/	159 (26%)	198 (32%)

Count (percentage) is reported in the table. Numbers are highlighted in red if the result was not reproduced during the datathon.

* Analysis was run in Python in this team. Analyses from the other teams were run in R.

/ Analysis/statistics was not considered.

Supplementary table 4. Numeric comparison of table 3 (all-cause deaths) in CAO/ARO/AIO-04 trial with team reproductions

	Original		Team 1		Team 2		Team 3		Team 4		Team 5*	
	Investiga-tional (n=613)	Control (n=623)	Investiga-tional (n=613)	Control (n=623)	Investiga-tional (n=613)	Control (n=623)	Investiga-tional (n=613)	Control (n=623)	Investiga-tional (n=613)	Control (n=623)	Investiga-tional (n=613)	Control (n=623)
**All-cause deaths**	96 (16%)	106 (17%)	96 (16%)	106 (17%)	96 (16%)	106 (17%)	96 (16%)	106 (17%)	/	/	96 (16%)	106 (17%)
**Rectal cancer**	54 (9%)	69 (11%)	54 (9%)	69 (11%)	54 (9%)	69 (11%)	54 (9%)	69 (11%)	62	69	54 (9%)	69 (11%)
**Toxicity from neoadjuvant or adjuvant chemotherapy**	8 (1%)	3 (<1%)	8 (1%)	3 (<1%)	8 (1%)	3 (0%)	8 (1%)	3 (<1%)	/	/	8 (1%)	3 (<1%)
**Postoperative death within 60 days after surgery**	4 (<1%)	6 (1%)	4 (1%)	6 (1%)	4 (1%)	6 (1%)	4 (<1%)	6 (1%)	/	/	4 (<1%)	6 (1%)
**Secondary malignancy**	3 (<1%)	1 (<1%)	3 (<1%)	1 (<1%)	3 (0%)	1 (0%)	3 (<1%)	1 (<1%)	/	/	3 (<1%)	1 (<1%)
**Intercurrent disease**	20 (3%)	17 (3%)	20 (3%)	17 (3%)	20 (3%)	17 (3%)	20 (3%)	17 (3%)	/	/	20 (3%)	17 (3%)
**Unknown or missing**	7 (1%)	10 (2%)	7 (1%)	10 (2%)	7 (1%)	9 (1%)	7 (1%)	9 (1%)	/	/	7 (1%)	10 (2%)

Count (percentage) is reported in the table. Numbers are highlighted in red if the result was not reproduced during the datathon.

* Analysis was run in Python in this team. Analyses from the other teams were run in R.

/ Analysis/statistics was not considered.

Supplementary table 5. Evaluation on the reproduced paper according to CONSORT 2010 checklist

CONSORT 2010 checklist of information to include when reporting a randomised trial*

Section/Topic	Item No	Checklist item	Evaluation
**Title and abstract**			
	1a	Identification as a randomised trial in the title	Reported
	1b	Structured summary of trial design, methods, results, and conclusions (for specific guidance see CONSORT for abstracts)	Reported
**Introduction**			
Background and objectives	2a	Scientific background and explanation of rationale	Reported
	2b	Specific objectives or hypotheses	Reported
**Methods**			
Trial design	3a	Description of trial design (such as parallel, factorial) including allocation ratio	Reported
	3b	Important changes to methods after trial commencement (such as eligibility criteria), with reasons	Not applicable
Participants	4a	Eligibility criteria for participants	Reported
	4b	Settings and locations where the data were collected	Reported
Interventions	5	The interventions for each group with sufficient details to allow replication, including how and when they were actually administered	Reported
Outcomes	6a	Completely defined pre-specified primary and secondary outcome measures, including how and when they were assessed	Reported
	6b	Any changes to trial outcomes after the trial commenced, with reasons	Not applicable
Sample size	7a	How sample size was determined	Reported but incomplete
	7b	When applicable, explanation of any interim analyses and stopping guidelines	Reported

Randomisation:			
Sequence generation	8a	Method used to generate the random allocation sequence	Reported
	8b	Type of randomisation; details of any restriction (such as blocking and block size)	Reported
Allocation concealment mechanism	9	Mechanism used to implement the random allocation sequence (such as sequentially numbered containers), describing any steps taken to conceal the sequence until interventions were assigned	Reported
Implementation	10	Who generated the random allocation sequence, who enrolled participants, and who assigned participants to interventions	Reported
Blinding	11a	If done, who was blinded after assignment to interventions (for example, participants, care providers, those assessing outcomes) and how	Not applicable
	11b	If relevant, description of the similarity of interventions	Not applicable
Statistical methods	12a	Statistical methods used to compare groups for primary and secondary outcomes	Reported
	12b	Methods for additional analyses, such as subgroup analyses and adjusted analyses	Reported
**Results**			
Participant flow (a diagram is strongly recommended)	13a	For each group, the numbers of participants who were randomly assigned, received intended treatment, and were analysed for the primary outcome	Reported
	13b	For each group, losses and exclusions after randomisation, together with reasons	Reported
Recruitment	14a	Dates defining the periods of recruitment and follow-up	Reported
	14b	Why the trial ended or was stopped	Not applicable
Baseline data	15	A table showing baseline demographic and clinical characteristics for each group	Reported
Numbers analysed	16	For each group, number of participants (denominator) included in each analysis and whether the analysis was by original assigned groups	Reported
Outcomes and estimation	17a	For each primary and secondary outcome, results for each group, and the estimated effect size and its precision (such as 95% confidence interval)	Unclear reporting for

			secondary outcomes
	17b	For binary outcomes, presentation of both absolute and relative effect sizes is recommended	Reported
Ancillary analyses	18	Results of any other analyses performed, including subgroup analyses and adjusted analyses, distinguishing pre-specified from exploratory	Reported
Harms	19	All important harms or unintended effects in each group (for specific guidance see CONSORT for harms)	Reported
**Discussion**			
Limitations	20	Trial limitations, addressing sources of potential bias, imprecision, and, if relevant, multiplicity of analyses	Potential bias not reported
Generalisability	21	Generalisability (external validity, applicability) of the trial findings	Reported
Interpretation	22	Interpretation consistent with results, balancing benefits and harms, and considering other relevant evidence	Reported
**Other information**			
Registration	23	Registration number and name of trial registry	Reported
Protocol	24	Where the full trial protocol can be accessed, if available	Broken link
Funding	25	Sources of funding and other support (such as supply of drugs), role of funders	Supply of drugs not reported

Citation: Schulz KF, Altman DG, Moher D, for the CONSORT Group. CONSORT 2010 Statement: updated guidelines for reporting parallel group randomised trials. BMC Medicine. 2010;8:18.

© 2010 Schulz et al. This is an Open Access article distributed under the terms of the Creative Commons Attribution License (http://creativecommons.org/licenses/by/2.0), which permits unrestricted use, distribution, and reproduction in any medium, provided the original work is properly cited.

*We strongly recommend reading this statement in conjunction with the CONSORT 2010 Explanation and Elaboration for important clarifications on all the items. If relevant, we also recommend reading CONSORT extensions for cluster randomised trials, non-inferiority and equivalence trials, non-pharmacological treatments, herbal interventions, and pragmatic trials. Additional extensions are forthcoming: for those and for up-to-date references relevant to this checklist, see www.consort-statement.org.
